# Antimicrobial susceptibility and genetic mechanisms of resistance of *Ureaplasma* isolates in North America between 2012 and 2023

**DOI:** 10.1128/aac.01868-24

**Published:** 2025-05-05

**Authors:** Joshua T. Waites, Donna M. Crabb, Amy E. Ratliff, Ken B. Waites, Li Xiao

**Affiliations:** 1 Departments of Pathology, The University of Alabama at Birmingham 189178, Birmingham, Alabama, USA; 2 Department of Medicine, The University of Alabama at Birmingham 164494, Birmingham, Alabama, USA; University of California San Francisco, San Francisco, California, USA

**Keywords:** *Ureaplasma*, susceptibility, mechanisms of resistance, erythromycin, tetracyclines, levofloxaxin

## Abstract

We analyzed antimicrobial susceptibilities of 415 *Ureaplasma* isolates from various sample types derived from different regions of the United States and Canada from 2012 to 2023 and investigated the genetic mechanisms of antimicrobial resistance. Minimum inhibitory concentration (MIC) ranges for erythromycin, tetracycline, and levofloxacin were 0.063–256, 0.016–64, and 0.063–32 µg/mL, respectively. MIC_50_ values for erythromycin, tetracycline, and levofloxacin were 2, 0.25, and 1 µg/mL, and MIC_90_ values were 4, 1, and 2 µg/mL, respectively. According to Clinical and Laboratory Standards Institute breakpoints, there were 61 (14.7%) isolates resistant to one or more drugs, and resistance rates for erythromycin, tetracycline, and levofloxacin were 2.4% (10/415), 6.5% (27/413), and 6.7% (28/415), respectively. Four isolates (1.0%) were resistant to two drugs. Mutations in domain V of 23S rRNA, mainly A2058G (*Escherichia coli* numbering), and/or in the *rpl*D gene encoding ribosomal protein L4 were identified in most erythromycin-resistant isolates. *Tet*(M) was detected in all isolates with tetracycline MIC ≥4 µg/mL but absent in 64.7% (11/17) of isolates with MIC of 2 µg/mL. For fluoroquinolone-resistant isolates, C248T (S83L) and G259A (E87K) mutations in *parC* were identified in most cases (23/26). In summary, erythromycin, tetracycline, and levofloxacin are still effective *in vitro* against most *Ureaplasma* isolates in North America.

## INTRODUCTION

*Ureaplasma* species are implicated as pathogens in nongonococcal urethritis, endometritis, chorioamnionitis, spontaneous abortion, and urinary calculi in adults, and prematurity, low birth weight, bacteremia, meningitis, and lung infections in neonates. These organisms are also implicated in systemic infections in immunosuppressed hosts, particularly joint infections in persons with hypogammaglobulinemia and as a cause of hyperammonemia in lung and kidney transplant recipients (1–7).

Macrolides, tetracyclines, and fluoroquinolones are the three main antimicrobial classes potentially useful in treating *Ureaplasma* infections (1). However, acquired resistance to drugs in each of these classes and the molecular mechanisms responsible for such resistance are well documented in *Ureaplasma* species, potentially limiting their use in some circumstances (8–12). Mechanisms for macrolide resistance are mainly attributed to the mutations in 23S rRNA and ribosomal proteins L4 and L22 that interfere with macrolide binding to the 50S ribosome, which eventually inhibits protein synthesis (8, 10, 13). The only known mechanism of naturally occurring resistance to tetracyclines in *Ureaplasma* species is mediated by *tet*(M), which prevents drug binding at the 30S ribosome (8). Fluoroquinolone resistance in *Ureaplasma*s is primarily mediated by mutations in the quinolone resistance-determining region (QRDR) of DNA topoisomerase IV (encoded by *parC* and *parE*) and DNA gyrase (encoded by *gyrA and gyrB)* (8, 9, 12).

*In vitro* antimicrobial susceptibility data for *Ureaplasma* species are extremely limited in the United States. Despite the availability of standardized methods for phenotypic measurement of MICs for *Ureaplasma* species (14), the dearth of susceptibility data is primarily because few physicians request MIC testing; the procedures are performed in very few reference laboratories; and there are no FDA-cleared commercial MIC test systems for *Ureaplasma*s available for use.

The University of Alabama at Birmingham (UAB) Diagnostic *Mycoplasma* Laboratory is a reference facility that performs MIC measurements on *Ureaplasma* species isolated from clinical specimens from all over the United States and Canada. Antibiograms with MIC summaries are made available at periodic intervals to clinicians, hospitals, and other laboratories that utilize our facility for diagnostic testing to guide empiric antimicrobial therapy pending culture and susceptibility testing results. We have summarized MICs performed for clinical diagnostic purposes for erythromycin, tetracycline, and levofloxacin on a collection of *Ureaplasma* isolates obtained from various specimen types over a 12-year period (2012–2023) and determined resistance mechanisms of all isolates shown to be non-susceptible.

## MATERIALS AND METHODS

### *Ureaplasma* specimens and isolates

MIC results for 415 clinical isolates of *Ureaplasma* species that underwent antimicrobial susceptibility testing (AST) between 2012 and 2023 were retrieved from the laboratory database. Original specimens were obtained from adults and neonates and included urine (64), cervix/vagina (56), urethra (29), nasopharynx (29), trachea (27), and a variety of other fluids and tissues (18). No source was specified for 192 of them. Specimens were obtained from medical centers in 22 states located in all geographic regions of the United States (AL, AK, CA, CO, IL, IA, KY, LA, MD, MA, MI, MN, NC, NJ, OH, NM, PA, RI, TN, VA, TX, and WA) and one Canadian Province (SK). Several specimens were received from reference laboratories located in CA, MN, UT, and VA, but information regarding their state of origin was not available. In most cases, differentiation between *U. urealyticum* and *U. parvum* was not performed, as this is not necessary for clinical diagnostic purposes. All bacterial isolates obtained from clinical specimens in our reference laboratory are deidentified and assigned a unique laboratory accession number and stored in case they are needed for further testing. No patient identifiers are available for them once diagnostic testing is completed. Therefore, this is not considered human subject research, and no informed consent from patients from whom organisms were isolated in culture or other approvals were obtained.

### Antimicrobial susceptibility testing

Reagent grade powders of known potency (µg/mL) of erythromycin, tetracycline, and levofloxacin were purchased from either Sigma-Aldrich (St. Louis, MO) or Fischer Scientific (Hampton, NH) during the 12-year surveillance period and stored frozen at 20°C in a desiccator. Stock solutions of antimicrobials were prepared and diluted in accordance with published Clinical Laboratory Standards Institute (CLSI) guidelines (14). MICs were measured in 10 B Broth prepared at UAB using a formulation specified by CLSI and interpreted by CLSI guidelines (14). The range of dilutions tested for each drug was 0.008–256 µg/mL. *U. urealyticum* American Type Culture Collection (ATCC) 33175 strain was used for quality control. MIC breakpoints specified by the CLSI to determine susceptibility or resistance were as follows: erythromycin susceptible (S) ≤ 8 µg/mL, resistant (R) ≥ 16 µg/mL; tetracycline S ≤ 1, R ≥ 2 µg/mL; and levofloxacin S ≤ 2 µg/mL, R ≥ 4 µg/mL.

#### DNA isolation

There were 55 of the 61 isolates resistant to erythromycin, tetracycline, and/or levofloxacin available for molecular genetic analysis. Isolates were stored at −80°C until analyzed. DNA was isolated from 1 mL of the original stock cultures that were used for MIC measurements using the TGuide Smart Universal DNA Kit operated on the Tiangen TGuide S16 Nucleic Acid Extractor (Tiangen Bioteck, Beijing, China). Standard operational protocol was followed for bacterial samples. DNA was eluted in 120 μL Buffer TB.

#### PCR and sequencing

*Ureaplasma* species determination was performed for antimicrobial-resistant isolates by a real-time PCR assay (15). The *U. parvum* primers/probes target the nucleoside 2-deoxyribosyltransferase gene *UU063* (strain ATCC 700970) that is identical in *U. parvum* type strains. The *U. urealyticum* primers/probes target the GUMAP protein gene (*UUR10_0680* in strain ATCC 33699) that is conserved in all *U. urealyticum* type strains. Real-time PCR was performed on Roche LightCycler 480 II using LightCycler 480 Genotyping Master (Roche Diagnostics, Indianapolis, IN).

Traditional PCR assays were performed to amplify the genes associated with antimicrobial resistance on a Veriti 96-well thermocycler (Applied Biosystems, Foster City, CA) using AccuPrime Pfx (Thermo Fisher, Waltham, MA). Primers are shown in Table S1. For erythromycin resistance, the full length of the two 23S rRNA genes, ribosomal protein genes *rpl*D (L4) and *rpl*V (L22) were amplified. For tetracycline resistance, PCRs were performed to evaluate the presence of *tet*(M), *tet*(O), *tet*(S), and *tet*(W) (16, 17). The full length of two 16S rRNA genes was also examined. For levofloxacin resistance, the QRDR region of *gyrA, gyrB*, *parC,* and *parE* genes was amplified. The two *parC*/*parE* operons in *U. urealyticum* isolates (Fig. S1) were differentiated by two additional PCRs if a sequence variation with mixed peaks in the Sanger sequencing trace file was identified. PCR amplicons were sequenced by the Sanger technique with both forward and reverse directions at the UAB Heflin Genomic Core. Sequences were analyzed using CLC Main Workbench 24 (Qiagen, Germantown, MD). The sequences were compared to the corresponding genes in the 14 ATCC reference strains (serovars 1–14) to determine the mutations associated with antimicrobial resistance. *U. urealyticum* serovar 9 (ATCC 33175), which harbors the *tet*(M) sequence and is resistant to tetracycline, was used as the *tet*(M)-positive control.

### Statistical analysis

IBM SPSS 29.0 was used for statistical analysis. A normality test was performed on MIC distributions. Independent-samples Kruskal-Wallis Test was used to compare MICs among different years. The Chi-Squared test was used to compare the resistance rates among the years with Bonferroni corrections.

## RESULTS

### MIC summaries

**Fig 1:**
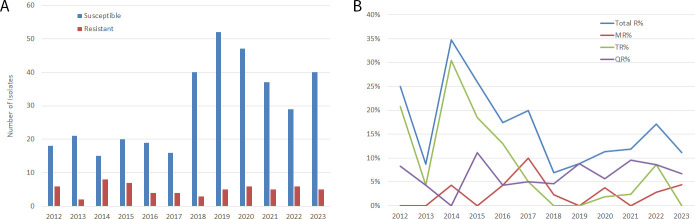
*Ureaplasma* isolates and resistance rates over the years. (**A**) Numbers of susceptible and resistant isolates over the 12 years. (**B**) Antimicrobial resistance rate over the years. MR, macrolide resistance rate; TR, tetracycline resistance rate; QR, quinolone resistance rate.

MIC summaries and distributions for each antimicrobial agent are shown in Table 1. MIC ranges for erythromycin, tetracycline, and levofloxacin were 0.063–256, 0.016–64, and 0.063–32 µg/mL, respectively. MIC_50_s for the three drugs were 2, 0.5, and 1 µg/mL, and MIC_90_s were 4, 1, and 2 µg/mL, respectively. The MICs of the three drugs were not normally distributed (*P* < 0.001) with a skewness to the lower susceptible ranges (Fig. S2). There were 61 (14.7%) isolates resistant to one or more drugs. Resistance rates for erythromycin, tetracycline, and levofloxacin were 2.4% (10/415), 6.5% (27/413), and 6.7% (28/415), respectively. Four isolates (0.8%) were resistant to two drugs. Over the 12-year study period, the overall resistance rate varied between 7.0% and 34.8% without a significant difference among the years (*P* = 0.073) (Fig. 1). The resistance rates for erythromycin (0%–10.0%) and fluoroquinolone (0%–11.1%) had no significant variations. However, the tetracycline resistance rate (0%–30.4%) was significantly higher in 2014 (30.4%).

**TABLE 1:** MIC summary and distribution for 415 *Ureaplasma* isolates^*a*^

Drug	No. (%) isolates with each MIC value (µg/mL)													MIC range (µg/mL)	MIC_50_ (µg/mL)	MIC_90_ (µg/mL)	% Susceptible
	0.016	0.032	0.063	0.125	0.25	0.5	1	2	4	8	16	32	>64				
Erythromycin (*n* = 415)	0	0	1	2	9	55	124	149	58	7	**2**	**2**	**6**	0.063–256	2	4	97.8
Tetracycline (*n* = 413)	1	11	20	78	105	111	60	**18**	**1**	**1**	**5**	**1**	**1**	0.016–64	0.25	1	93.5
Levofloxacin (*n* = 415)	0	0	6	11	42	132	146	50	**22**	**2**	**2**	**2**	**0**	0.063–32	1	2	93.3

^*a*^ There were 61 (14.7%) isolates resistant to one or more drugs; among them, four isolates (1.0%) were resistant to two drugs. Bold values indicate isolates considered resistant based on CLSI breakpoints (14).

### Resistance mechanisms

There were 55 of 61 (90%) isolates (28 *U*. *parvum*, 24 *U*. *urealyticum*, and 3 mixed species) with phenotypic resistance to one or more antimicrobial agents available for genetic analysis for the mechanisms of antimicrobial resistance (Table S2). Among them, four have been previously described (18). For the three isolates containing both species, it was not possible to determine which of them contained the mutations conferring antimicrobial resistance.

Ten isolates were resistant to erythromycin (MICs ≥ 16 µg/mL; 2 *U*. *parvum,* 8 *U*. *urealyticum)* (Table 2). Genetic alterations known to be associated with macrolide resistance in *Ureaplasma* or other *Mycoplasma* species were detected in seven isolates. A2058G (*E. coli* numbering) mutations in domain V of the 23S rRNA gene were found in five isolates, presenting as a single copy in four isolates and double copies in one isolate. All five isolates had high erythromycin MICs (256 µg/mL). Another *U. urealyticum* isolate also had a high MIC of 256 µg/mL and had insertions in both 23S rRNA genes: 2060_2061insA (*E. coli* numbering) in one copy and 2721_2722insTAG in the other copy. This isolate also had a duplication, 229_243dup (AAAGCACGTACAGGT), corresponding to amino acid change 77_81dup (KARTG) in the ribosomal protein L4 gene *rplD*. One *U. parvum* isolate (MIC = 32 µg/mL) had a deletion, 193_198del (TGAAGA), corresponding to amino acid change 65_66del (WR) in *rplD*. Three isolates with relatively lower MICs (16 or 32 µg/mL) had no genetic alterations previously shown to be associated with macrolide resistance detected. One of them (MIC = 16 µg/mL) had a T1598C (*E. coli* numbering) alteration in one 23S rRNA gene. No non-synonymous mutations were detected in the *rplV* gene in the 10 erythromycin-resistant isolates.

**TABLE 2:** Erythromycin-resistant *Ureaplasma* isolates and genetic alterations

Isolate	Year	Species	MIC (µg/mL)	23S rRNA op1	23S rRNA op2	*rplD* (aa)	*rplV* (aa)
63166^*a*^	2014	Uu	256	C1429A (1404)	**A2072G (2058**)	WT	A588G
69278	2017	Uu	256	**A2065G (2058**)	**A2065G (2058**)	WT	WT
70827	2017	Up	256	**A2065G (2058**)	WT	WT	WT
82821	2020	Uu	256	WT	**A2065G (2058**)	WT	WT
83293	2020	Uu	256	2731_2732insTAG (2721_2722)	**2067_2068insA (2060_2061**)	**229_243dupAAAGCACGTACAGGT (p.77_81dupKARTG**)	WT
90648	2022	Uu	256	T1594C (1562)	**A2065G (2058**)	WT	C138T
66934	2016	Up	32	WT	WT	**193_198delTGAAGA (p.65_66delWR**)	WT
73475	2018	Uu	32	WT	WT	WT	WT
91288	2023	Up	16	WT	WT	A249G	WT
93035	2023	Uu	16	T1630C (1598)	WT	WT	A588G

^*a*^ Described previously. Op: operon; (aa): amino acid changes; Uu: *Ureaplasma urealyticum*; Up: *Ureaplasma parvum;* WT: wild type. Numbers and letters in bold type refer to variations believed to be associated with macrolide resistance. Numbers in parentheses refer to the *Escherichia coli* numbering system.

**Fig 2:**
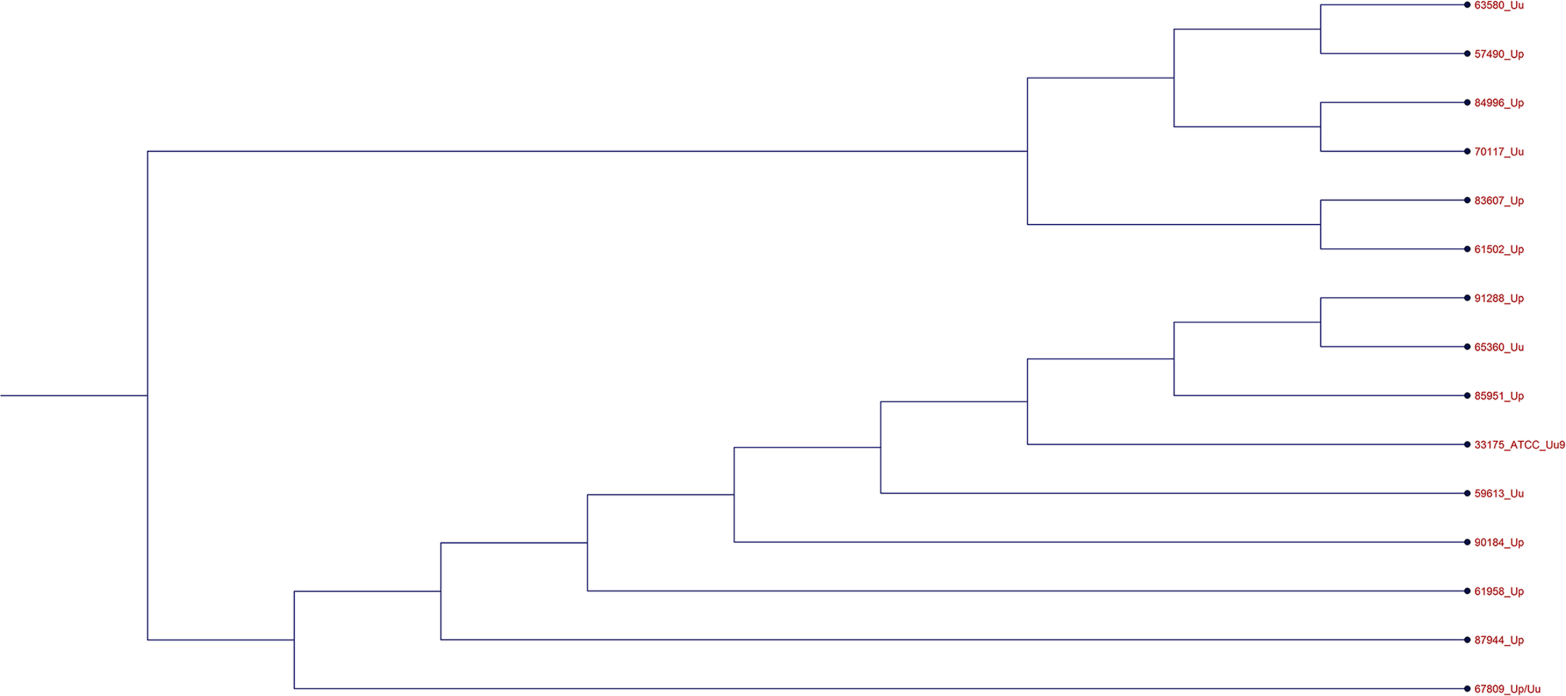
Phylogenetic tree of Tet(M) from *Ureaplasma* spp. isolates. The derived Tet(M) sequences were aligned, and a phylogenetic tree was constructed using the Neighbor Joining method. CLC Genomics 24 was used for alignment and tree construction.

Among 55 *Ureaplasma* isolates that demonstrated phenotypic resistance to one or more antimicrobial agents, which were available for genetic characterization, there were 23 isolates with tetracycline MICs ≥ 2 µg/mL designated as resistant (8 *U*. *parvum*, 14 *U*. *urealyticum*, and one mixed species) (14) (Table 3). The *tet*(M) transposon was detected in all six isolates with tetracycline MIC ≥4 µg/mL, but only in 6 (3 *U*. *parvum* and 3 *U*. *urealyticum*) out of 17 (35.3%) isolates with tetracycline MIC of 2 µg/mL. Testing the other 32 tetracycline-susceptible isolates found that *tet*(M) was also present in two isolates with MICs < 1 µg/mL. The derived 14 Tet(M) sequences can be classified into two major subgroups, and eight (group I) were grouped with the Tet(M) sequence from reference strain ATCC 33175 (Fig. 2). There was no correlation between sequence types and MIC values (*P* = 1.0, Mann-Whitney U test). PCR testing for the presence of *tet*(O), *tet*(S), or *tet*(W) sequences in all 55 drug-resistant isolates was negative. No known 16S rRNA mutations associated with tetracycline resistance were found in the 23 isolates. Four sequence variations were identified in three isolates (MIC = 2 µg/mL) with positions away from currently known tetracycline binding sites in 16S rRNA. Three variations, T662C, C717T, and G1368A, were found in two isolates that also had *tet*(M). One isolate without *tet*(M) had a G260A variation. The potential role of these variations in tetracycline resistance needs further investigation.

**TABLE 3:** Tetracycline-resistant *Ureaplasma* isolates and genetic alterations^*a*^

Isolate	Year	Species	MIC (µg/mL)	*tet*(M)	Tet(M) subgroup	16S rRNA op1	16S rRNA op2
59613	2013	Uu	64	Pos	II	WT	WT
57490	2012	Up	32	Pos	I	WT	WT
61958	2014	Up	16	Pos	II	WT	WT
90184	2022	Up	16	Pos	II	WT	WT
83607	2020	Up	8	Pos	I	WT	WT
67809^*b*^	2016	Up/Uu	4	Pos	II	WT	WT
58005	2012	Uu	2	Neg		WT	WT
58006	2012	Uu	2	Neg		WT	WT
58011	2012	Up	2	Neg		WT	G269A (260)
58026	2012	Uu	2	Neg		WT	WT
61502	2014	Up	2	Pos	I	WT	WT
63580	2014	Uu	2	Pos	I	WT	WT
63595	2014	Uu	2	Neg		WT	WT
65360	2015	Uu	2	Pos	II	C722T (717)	G1351A (1368)
65524^*b*^	2015	Uu	2	Neg		WT	WT
65944	2015	Uu	2	Neg		WT	WT
66107	2015	Uu	2	Neg		WT	WT
66346	2015	Uu	2	Neg		WT	WT
67423	2016	Uu	2	Neg		WT	WT
67514	2016	Uu	2	Neg		WT	WT
70117	2017	Uu	2	Pos	I	WT	T667C (662)
84996	2021	Up	2	Pos	I	WT	WT
87944	2022	Up	2	Pos	II	WT	WT

^*a*^ Only 23 of 27 tetracycline-resistant *Ureaplasma* isolates were available for genetic characterization studies.

^*b*^ Described previously. Op: operon; Uu: *Ureaplasma urealyticum*; Up: *Ureaplasma parvum;* WT: wild type; Pos: positive; Neg: negative. Numbers in parentheses refer to the *Escherichia coli* numbering system.

There were 26 isolates (18 *U*. *parvum*, 5 *U*. *urealyticum*, and 3 mixed species) with MICs ≥ 4 µg/mL, indicative of levofloxacin resistance based on the CLSI breakpoint (14) available for genetic evaluation (Table 4). The *parC* mutations C248T (S83L) and G259A (E87K), known to be associated with fluoroquinolone resistance in *Ureaplasma* species (9, 11, 19–26), were identified in 20 and 3 isolates, respectively. Four isolates with the ParC S83L mutation had additional mutations in *gyrB* (D443N, P462S, E482K, and E482G) and showed high levofloxacin MICs (≥ 16 µg/mL). Two isolates with an MIC of 4 µg/mL only had single *parE* mutations (D426N and V417T). One *U. urealyticum* isolate (65524) (MIC = 8 µg/mL) had multiple mutations in *gyrB*, *parC,* and *parE* genes, while none of them were in QRDR. On the other hand, plasmid-mediated quinolone resistance determinants qnrB, aac (6)−1b, and qepA were described previously in this isolate (18). This isolate was also resistant to tetracycline (MIC = 2 µg/mL) without a genetic mechanism identified. No QRDR *gyrA* mutations were identified in the 26 isolates.

**TABLE 4:** Levofloxacin-resistant *Ureaplasma* isolates and genetic alterations^*a*^

Isolate	Year	Species	MIC (µg/mL)	*gyrA* (aa)	*gyrB* (aa)	*parC* (aa)	*parE* (aa)
74044	2018	Uu	32	WT	**G1444A (E482K**)	**C248T (S83L**)	WT
80608	2019	Uu	32	WT	**G1327A (D443N**)	**C248T (S83L**)	WT
66278^*b*^	2015	Up	16	WT	**C1384T (P462S**)	**C248T (S83L),** G943A (V315I)	WT
90648	2022	Uu	16	WT	**A1445G (E482G**)	**C248T (S83L**)	T1353A, op II
65524^*b*^	2015	Uu	8	WT	G775A (G259N)	G2428A (V810I)	C250A (Q84K); A253G (T85A)
84773	2021	Up	8	WT	WT	**C248T (S83L**)	WT
58011	2012	Up	4	WT	WT	WT	**GT1249..1250AC (V417T**)
60153	2013	Up	4	WT	WT	**G259A (E87K**)	WT
64940	2015	Up	4	A135G	G1611A	**C248T (S83L**)	WT
71094	2017	Up/Uu	4	WT	WT	**C248T (S83L**)	WT
67809^*b*^	2016	Up/Uu	4	WT	WT	**C248T (S83L**)	WT
80469	2019	Up/Uu	4	WT	WT	T96C; **C248T (S83L**)	WT
80103	2019	Up	4	WT	WT	**C248T (S83L**)	WT
76854	2019	Up	4	WT	WT	**C248T (S83L**)	WT
80584	2019	Up	4	WT	WT	**C248T (S83L**)	WT
82226	2020	Up	4	WT	WT	**C248T (S83L**)	WT
81945	2020	Up	4	WT	WT	**C248T (S83L**)	WT
82050	2020	Up	4	WT	WT	**C248T (S83L**)	WT
85534	2021	Up	4	WT	WT	**G259A (E87K**)	WT
84534	2021	Up	4	WT	A1221G	**G259A (E87K**)	WT
85951	2021	Up	4	WT	WT	**C248T (S83L**)	WT
90454	2022	Up	4	WT	WT	**C248T (S83L**)	WT
90056	2022	Up	4	WT	WT	**C248T (S83L**)	WT
91820	2023	Uu	4	WT	WT	WT	**G1279A (D426N), op II**
91158	2023	Up	4	WT	WT	**C248T (S83L**)	WT
91241	2023	Up	4	WT	WT	**C248T (S83L**)	WT

^*a*^ Only 26 of 28 levofloxacin-resistant *Ureaplasma* isolates were available for genetic characterization studies.

^*b*^ Described previously. Op: operon; Uu: *Ureaplasma urealyticum*; Up: *Ureaplasma parvum;* (aa): amino acid changes; WT: wild type; Numbers and letters in bold type refer to variations believed to be associated with fluoroquinolone resistance. Numbers in parentheses refer to the *Escherichia coli* numbering system.

Among the 55 genetically evaluated isolates, four isolates were resistant to two drugs. *U. urealyticum* isolate (90648) was highly resistant to erythromycin (MIC = 256 µg/mL) and levofloxacin (MIC = 16 µg/mL), due to the presence of multiple mutations in 23S rRNA (A2058G), ParC (S83L), and GyrB (E482G).

## DISCUSSION

We have performed the largest *in vitro* susceptibility survey of *Ureaplasma* clinical isolates from various geographic regions in the United States described to date and investigated the mechanisms of resistance to the three main antimicrobial classes used for treating infections due to these organisms. More than 85% of these clinical isolates were susceptible to all three drug classes tested, and only 1.0% were resistant to more than one drug class. Molecular mechanisms known to be associated with phenotypic resistance were identified in most of the resistant isolates that were evaluated.

The most recent large-scale *in vitro* susceptibility survey for *Ureaplasma* species in the United States, published by Fernandez et al. (25), included 250 isolates collected during 2015–2016, but the geographic distribution of the sources of these isolates was not reported. That study reported a levofloxacin resistance rate of 6.4% for *U. parvum* and 5.2% for *U. urealyticum*, similar to our findings of 6.5% levofloxacin resistance. However, these investigators found no macrolide resistance and only a single isolate of *U. parvum* with resistance to tetracycline (MIC = 8 µg/mL), which was also resistant to levofloxacin (MIC = 4 µg/mL). By contrast, a 2017 report from Florida in which 73 *Ureaplasma* isolates obtained from urine specimens collected from female college students found only 1.6% resistance to tetracycline and levofloxacin and 0% for erythromycin (27). A survey from France conducted between 2010 and 2015 that utilized CLSI guidelines for MIC determinations included susceptibility data for 831 *Ureaplasma* isolates. They reported tetracycline and levofloxacin resistance rates of 7.5% and 1.2%, respectively (11). The *tet*(M) gene was found in all tetracycline-resistant isolates, and mutations in the *parC* or *parE* genes were found in all levofloxacin-resistant isolates. Isolates that were moxifloxacin-resistant also had a mutation in the *gyrA* gene.

Interestingly, Fernandez et al. (25) detected *tet*(M) in the single *U. parvum* that had an elevated MIC for tetracycline, but also in 9 *U*. *parvum* isolates that had low MICs within the susceptible range. Other investigators have noted that *tet*(M) can often be found in isolates (mainly *U. parvum*) with phenotypically low tetracycline MICs (28). On the other hand, Pereyre et al. showed that six *U. urealyticum* isolates with MICs of 2 and 4 µg/mL did not harbor *tet*(M) (29). Our study detected *tet*(M) in all isolates with tetracycline MIC ≥4 µg/mL but was absent in 11 out of 17 (64.7%) isolates with MICs of 2 µg/mL, which would be considered resistant based on current CLSI breakpoints (14). Moreover, *tet*(M) was detected in two isolates with MICs < 1 µg/mL. Previous and current findings suggest that actual MIC determination is necessary to document tetracycline resistance in *Ureaplasma* species. Merely testing for the presence of *tet*(M) will result in either overestimation of tetracycline resistance due to its presence in fully susceptible isolates with low MICs or underestimation of tetracycline resistance due to its absence in isolates with MICs slightly above the CLSI breakpoint (two or 4 µg/mL). Dumke reported that the *tet*(M) detection rate could be about 10-fold of the actual tetracycline resistance rate (42.9% vs 4.1%) in *Ureaplasma* species (21). In this study, 10 of the 11 isolates with an

MIC of 2 µg/mL lacking a *tet*(M) sequence were *U. urealyticum*. Whether there are correlations regarding the existence of the *tet*(M) sequence between the two *Ureaplasma* species and tetracycline susceptibility is worth further investigation. Alternatively, since all isolates in this study with MICs ≥ 4 µg/mL carried *tet*(M)*,* it might be reasonable to raise the breakpoint for resistance by one dilution from 2 to 4 µg/mL.

There are reports from various countries in recent years that describe very high rates of antimicrobial resistance in *Ureaplasma* species (20, 26, 30, 31). However, some of these studies used commercial MIC kits that are not compliant with CLSI methods or interpretive breakpoints for susceptibility and resistance and tend to overestimate resistance (11, 32). Thus, their results cannot be compared directly with other surveys that adhered to CLSI procedures. Moreover, resistance rates are expected to be different in various countries because antimicrobial consumption can be quite different, and this can help drive the development of antimicrobial resistance, as is typical in some Asian countries such as China. Some studies have been able to document resistance by identifying the molecular mechanisms responsible (20, 24, 26).

We have determined the mechanisms of resistance to the three drugs in most of the isolates. Data from this study proved again that the presence of only one copy of the A2058G mutation in 23S rRNA is sufficient to convey high-level erythromycin resistance in *Ureaplasma*s (observed in four isolates), while mutations in ribosomal proteins cause relatively low-level resistance, similar to what was reported in previous studies (10, 33). Mutations in the 16S rRNA gene associated with tetracycline resistance have been identified in other *Mycoplasma* species (34, 35). However, no such mutations were noted in *Ureaplasma* species in the current and previous studies. Carrying a *tet*(M) sequence explains tetracycline resistance with higher MICs in *Ureaplasma* species, while contradictions exist in isolates with lower MICs. This phenomenon may be partially due to the complex mechanisms of *tet*(M) expression regulation, such as transcriptional attenuation or small RNA-mediated regulation, which is present in *Enterococcus faecalis* and *E. faecium* and may exist in *M. hominis* (36–38). However, there are no data regarding factors that may influence (*tet*)M expression in ureaplasmas.

Our study showed that fluoroquinolone resistance in *Ureaplasma* spp. is mainly caused by mutations (S83L or E87K) in the ParC subunit of DNA topoisomerase IV (23/26, 88.5%). Mutations in the ParE subunit of DNA topoisomerase IV and GyrA or GyrB of DNA gyrase in the QRDRs are known to be less common but can potentiate the resistance together with ParC mutations. We found two *U. urealyticum* isolates with high-level levofloxacin resistance (MICs 16–32 µg/mL) had mutations S83L in ParC and at position 482 in GyrB, similar to what was reported in an isolate from Serbia by Boostrom et al. (12).

Fourteen *Ureaplasma* isolates in this study had elevated MICs to erythromycin (three isolates) or tetracycline (11 isolates) but lacked a known molecular mechanism to account for the resistance. It is possible that other mechanisms could be operable in these organisms, such as intrinsic active efflux or resistance genes carried by mobile elements. Analysis of their complete genome sequences may provide insights into which other mechanisms may be occurring in these cases. Whether the resistance detected in 61 isolates was driven by selective antimicrobial pressure and/or related to host immune status is not known because clinical histories or diagnoses of the patients were not accessible.

In general, most *in vitro* susceptibility studies of *Ureaplasma* species from the United States and Europe published over the past decade, including the present one, that have utilized CLSI guidelines for measurement of MICs have found that resistance to commonly used agents is still relatively low. This suggests macrolides, tetracyclines, and fluoroquinolones should still be effective against *Ureaplasma* infections. However, using doxycycline post-exposure prophylaxis for bacterial sexually transmitted infection prevention (39) raises a concern of increased resistance to tetracycline drugs in *Ureaplasma* species. Continued monitoring of antimicrobial resistance is needed, and due to the potential severity of opportunistic systemic infections caused by *Ureaplasma* species in immunosuppressed hosts, performance of *in vitro* susceptibility testing in this setting or in instances where treatment with first-line drugs has failed is recommended.

## SUPPLEMENTAL MATERIAL

Supplemental material aac.01868-24-s0001.pdf Tables S1 and S2; Fig. S1 and S2.

ASM does not own the copyrights to Supplemental Material that may be linked to, or accessed through, an article. The authors have granted ASM a non-exclusive, world-wide license to publish the Supplemental Material files. Please contact the corresponding author directly for reuse.
