# Supplementary material for: Antimicrobial susceptibility and genetic mechanisms of resistance of Ureaplasma isolates in North America between 2012 and 2023
Source: Antimicrob Agents Chemother. 2025 May 5;69(6):e01868-24. doi: 10.1128/aac.01868-24 (PMC12135515; doi:10.1128/aac.01868-24)
Supplement: Supplemental material — Tables S1 and S2; Fig. S1 and S2. [file aac.01868-24-s0001.pdf]

Supplementary Table 1. Primers Used for PCR Assays to Detect Resistance Genes

Target	Primer Name	Sequence (5'- to 3'-)	Purpose	Reference
Erythromycin resistance:				
Up 23S rRNA operon I	Usp23S-F	ATGGTCGGATTCTATTTAGTT	PCR/sequencing	This study
	Up23S-op1R	ATTCGTCTTCTGGTGTTT	PCR/sequencing	This study
Up 23S rRNA operon II	Usp23S-F	ATGGTCGGATTCTATTTAGTT	PCR/sequencing	This study
	Up23S-op2R	GCTGCTTGTTCATTTTTTG	PCR/sequencing	This study
Uu 23S rRNA operon I	Usp23S-F	ATGGTCGGATTCTATTTAGTT	PCR/sequencing	This study
	Uu23S-op1R	ATGGAAGTTTATTGTTTTTGGA	PCR/sequencing	This study
Uu 23S rRNA operon II	Usp23S-F	ATGGTCGGATTCTATTTAGTT	PCR/sequencing	This study
	Uu23S-op2R	GCGTCTTGTAATTGTTTTGT	PCR/sequencing	This study
rplD	Usp-L4-F	TCGTAAGCGATCCACAAG	PCR/sequencing	This study
	Usp-L4-R	TGAAGTACGAATTTTTGCAGG	PCR/sequencing	This study
rplV	Usp-L22-F	CAAAAACCATACAGAAGCTAAAC	PCR/sequencing	This study
	Usp-L22-R	CTACATGATCAATACCAGCA	PCR/sequencing	This study
Tetracycline resistance:				
Up 16S rRNA operon I	Up-16S-op1-F	CTTGACATTTTCTACACTTTTTT	PCR/sequencing	This study
	Usp-16S-R	AAACTAAATAGAATCCGACCA	PCR/sequencing	This study
Up 16S rRNA operon II	Up-16S-op2-F	TGAACAAGCAACACGAAT	PCR/sequencing	This study
	Usp-16S-R	AAACTAAATAGAATCCGACCA	PCR/sequencing	This study
Uu 16S rRNA operon I	Uu-16S-op1-F	AAGGCCTAAAATACTCAAAAATAA	PCR/sequencing	This study
	Usp-16S-R	AAACTAAATAGAATCCGACCA	PCR/sequencing	This study
Uu 16S rRNA operon II	Uu-16S-op2-F	TCCCCCACTTTTTTTGTTT	PCR/sequencing	This study
	Usp-16S-R	AAACTAAATAGAATCCGACCA	PCR/sequencing	This study
tet(M)	tet-1	GCTCAYGTTGAYGCAGGAA	PCR/sequencing	Barbosa et al., 1999
	tet-2	AGGATTTGGCGGSACTTCKA	PCR/sequencing	Barbosa et al., 1999
	TetM-F	TTATCAACGGTTTATCAGG	PCR/sequencing	Blanchard et al., 1992
	TetM-R	CGTATATATGCAAGACG	PCR/sequencing	Blanchard et al., 1992
tet(O)	tetO-F	AACTTAGGCATTCTGGCTCAC	PCR/sequencing	Wang et al., 2017
	tetO-R	CTATGGACAACCCGACAGAAG	PCR/sequencing	Wang et al., 2017
tet(S)	tetS-F	TAGATACTCCTGGACACAT	PCR/sequencing	Wang et al., 2017
	tetS-R	ATGAGAATGACCTCGTTAC	PCR/sequencing	Wang et al., 2017
tet(W)	tetW-F	CGGATTGTGGCATTTGT	PCR/sequencing	Wang et al., 2017
	tetW-R	GCATAGAGGGTGAAGGAG	PCR/sequencing	Wang et al., 2017
Levofloxaxin resistance:				
gryB-gyrA	gyrB-CF	GTGAAGTTCGTCCTTATGTTAAT	PCR/sequencing	This study

	gyrA-2	CTGATGGTAAAACACTTGG	PCR/sequencing	Bebear et al., 2000
	gyrB-CR	ATTTTTAGGATCCATTGTTGTTTC	Sequencing	This study
	gyrA-F	AACAATGGATCCTAAAAA	Sequencing	This study
parE-parC	parE-CF2	TGCTCGTGAAGAAACTAAAA	PCR/sequencing	This study
	parC-6	CACTATCATCAAAGTTTGGAC	PCR/sequencing	Bebear et al., 2000
	parE-CR1	TTATTTAGACTCCTCATTATTGATT	Sequencing	This study
	parC-CF	GGGTAATGACGTAAGTATTCGA	Sequencing	Xiao et al., 2012
Uu *parC* operon I	Uu-parC-F	ACAAGCTGAACGTCAAATCAA	PCR/sequencing	This study
	Uu-parC-op1-R	CAAAAAAGCGCTACAACCC	PCR/sequencing	This study
Uu *parC* operon II	Uu-parC-F	ACAAGCTGAACGTCAAATCAA	PCR/sequencing	This study
	Uu-parC-op2-R	CATTCAAAAAAGCGCCACA	PCR/sequencing	This study

References Cited in Supplemental Table 1:

Barbosa TM, Scott KP, Flint, HJ. 1999. Evidence for recent intergeneric transfer of a new tetracycline resistance gene, *tet*(W), isolated from *Butyrivibrio fibrisolvens*, and the occurrence of *tet*(O) in ruminal bacteria. Environmental Microbiology **1**:53–64.

Bebear CM, Grau O, Charron A, Renaudin H, Gruson D, Bebear C. 2000. Cloning and nucleotide sequence of the DNA gyrase (*gyrA*) gene from *Mycoplasma hominis* and characterization of quinolone-resistant mutants selected in vitro with trovafloxacin. Antimicrob Agents Chemother **44:** 2719-27.

Xiao L, Crabb DM, Duffy LB, Paralanov V, Glass JI, Waites KB. 2012. Chromosomal mutations responsible for fluoroquinolone resistance in *Ureaplasma* species in the United States. Antimicrob Agents Chemother 56:2780-2783.

Blanchard A, Crabb DM, Dybvig K, Duffy LB, Cassell GH. 1992. Rapid detection of *tet*M in *Mycoplasma hominis* and *Ureaplasma urealyticum* by PCR: *tet*M confers resistance to tetracycline but not necessarily to doxycycline. FEMS Microbiol Lett **74:**277-281. Blanchard, et al., FEMS Microbiol Lett 1992;74:277.

Wang N, Hang X, Zhang M, Liu X, Yang H. 2017. Analysis of newly detected tetracycline resistance genes and their flanking sequences in human intestinal bifidobacteria. Sci Rep **7:**6267.

Supplementary Table 2. Summary of antimicrobial resistant *Ureaplasma* isolates

Isolate	Year	Species	MIC (µg/mL)			23S rRNA op1	23S rRNA op2	*rplD* (aa)	*rplV* (aa)	*tet*(M)	Tet(M) subgroup	16S rRNA op1	16S rRNA op2	*gyrA* (aa)	*gyrB* (aa)	*parC* (aa)	*parE* (aa)
			ERY	TET	LEVO												
57490	2012	Up	2	**32**	1					**Pos**	I	WT	WT				
58005	2012	Uu	4	**2**	2					Neg		WT	WT				
58006	2012	Uu	4	**2**	2					Neg		WT	WT				
58011	2012	Up	4	**2**	4					Neg		WT	G269A (260)	WT	WT	WT	**GT1249..1250AC (V417T)**
58026	2012	Uu	2	**2**	2					Neg		WT	WT				
59613	2013	Uu	1	**64**	1					**Pos**	II	WT	WT				
60153	2013	Up	1	0.25	**4**					Neg				WT	WT	**G259A (E87K)**	WT
61502	2014	Up	0.5	**2**	0.5					**Pos**	I	WT	WT				
61958	2014	Up	2	**16**	2					**Pos**	II	WT	WT				
63166*	2014	Uu	**256**	0.5	1	C1429A (1404)	A2072G (2058)	WT	A588G	Neg							
63580	2014	Uu	2	**2**	2					**Pos**	I	WT	WT				
63595	2014	Uu	1	**2**	2					Neg		WT	WT				
64940	2015	Up	2	0.02	**4**					Neg				A135G	G1611A	**C248T (S83L)**	WT
65360	2015	Uu	4	**2**	2					**Pos**	II	C722T (717)	G1351A (1368)				
65524*	2015	Uu	2	**2**	**8**					Neg		WT	WT	WT	G775A (G259N)	G2428A (V810I)	C250A (Q84K); A253G (T85A)
65944	2015	Uu	4	**2**	2					Neg		WT	WT				
66107	2015	Uu	4	**2**	2					Neg		WT	WT				
66278*	2015	Up	2	0.13	**16**					Neg				WT	**C1384T (P462S)**	**C248T (S83L),** G943A (V315I)	WT
66346	2015	Uu	4	**2**	2					Neg		WT	WT				
66934	2016	Up	**32**	0.13	0.5	WT	WT	193_198delTGAAGA (p.65_66delWR)	WT	Neg							
67423	2016	Uu	4	**2**	2					Neg		WT	WT				
67514	2016	Uu	4	**2**	1					Neg		WT	WT				
67809*	2016	Up/Uu	1	**4**	**4**					**Pos**	II	WT	WT	WT	WT	**C248T (S83L)**	WT
69278	2017	Uu	**256**	1	2	A2065G (2058)	A2065G (2058)	WT	WT	Neg							
70117	2017	Uu	2	**2**	1					**Pos**	I	WT	T667C (662)				
70827	2017	Up	**256**	0.13	1	A2065G (2058)	WT	WT	WT	Neg							

71094	2017	Up/Uu	1	0.06	**4**					Neg				WT	WT	**C248T (S83L)**	WT
73475	2018	Uu	**32**	0.5	2	WT	WT	WT	WT	Neg							
74044	2018	Uu	2	1	**32**					Neg				WT	**G1444A (E482K)**	**C248T (S83L)**	WT
76854	2019	Up	1	0.13	**4**					Neg				WT	WT	**C248T (S83L)**	WT
80103	2019	Up	1	0.25	**4**					Neg				WT	WT	**C248T (S83L)**	WT
80469	2019	Up/Uu	1	0.5	**4**					Neg				WT	WT	T96C; **C248T (S83L)**	WT
80584	2019	Up	0.5	0.13	**4**					Neg				WT	WT	**C248T (S83L)**	WT
80608	2019	Uu	0.5	0.13	**32**					Neg				WT	**G1327A (D443N)**	**C248T (S83L)**	WT
81945	2020	Up	2	0.25	**4**					Neg				WT	WT	**C248T (S83L)**	WT
82050	2020	Up	1	0.25	**4**					Neg				WT	WT	**C248T (S83L)**	WT
82226	2020	Up	1	0.5	**4**					Neg				WT	WT	**C248T (S83L)**	WT
82821	2020	Uu	**256**	0.5	2	WT	A2065G (2058)	WT	WT	Neg							
83293	2020	Uu	**256**	0.13	0.5	2731_2732insTAG (2721_2722)	2067_2068insA (2060_2061)	229_243dupAAAGCACGTACAGGT (p.77_81dupKARTG)	WT	Neg							
83607	2020	Up	4	**8**	0.5					**Pos**	I	WT	WT				
84534	2021	Up	1	0.13	**4**					Neg				WT	A1221G	**G259A (E87K)**	WT
84773	2021	Up	4	0.25	**8**					Neg				WT	WT	**C248T (S83L)**	WT
84996	2021	Up	1	**2**	0.25					**Pos**	I	WT	WT				
85534	2021	Up	1	0.25	**4**					Neg				WT	WT	**G259A (E87K)**	WT
85951	2021	Up	2	0.13	**4**					**Pos**	II			WT	WT	**C248T (S83L)**	WT
87944	2022	Up	2	**2**	0.5					**Pos**	II	WT	WT				
90056	2022	Up	2	0.13	**4**					Neg				WT	WT	**C248T (S83L)**	WT
90184	2022	Up	4	**16**	0.25					**Pos**	II	WT	WT				
90454	2022	Up	2	0.25	**4**					Neg				WT	WT	**C248T (S83L)**	WT
90648	2022	Uu	**256**	0.25	**16**	T1594C (1562)	A2065G (2058)	WT	C138T	Neg				WT	**A1445G (E482G)**	**C248T (S83L)**	T1353A, op II
91158	2023	Up	4	0.25	**4**					Neg				WT	WT	**C248T (S83L)**	WT
91241	2023	Up	8	0.25	**4**					Neg				WT	WT	**C248T (S83L)**	WT
91288	2023	Up	**16**	0.5	0.5	WT	WT	A249G	WT	**Pos**	II						
91820	2023	Uu	1	0.5	**4**					Neg				WT	WT	WT	**G1279A (D426N), op II**
93035	2023	Uu	**16**	1	1	T1630C (1598)	WT	WT	A588G	Neg							

Note: * Described previously. Ery, erythromycin; Tet, tetracycline; Lev, levofloxacin; Op, operon; Uu, *Ureaplasma urealyticum*; Up, *Ureaplasma parvum;* (aa), amino acid changes; WT, wild type. Numbers and letters in bold type refer to variations believed to be associated with macrolide resistance. Numbers in parentheses refer to *Escherichia coli* numbering system.

Supplementary Figure 1. *parE-parC* operons in *U. urealyticum*

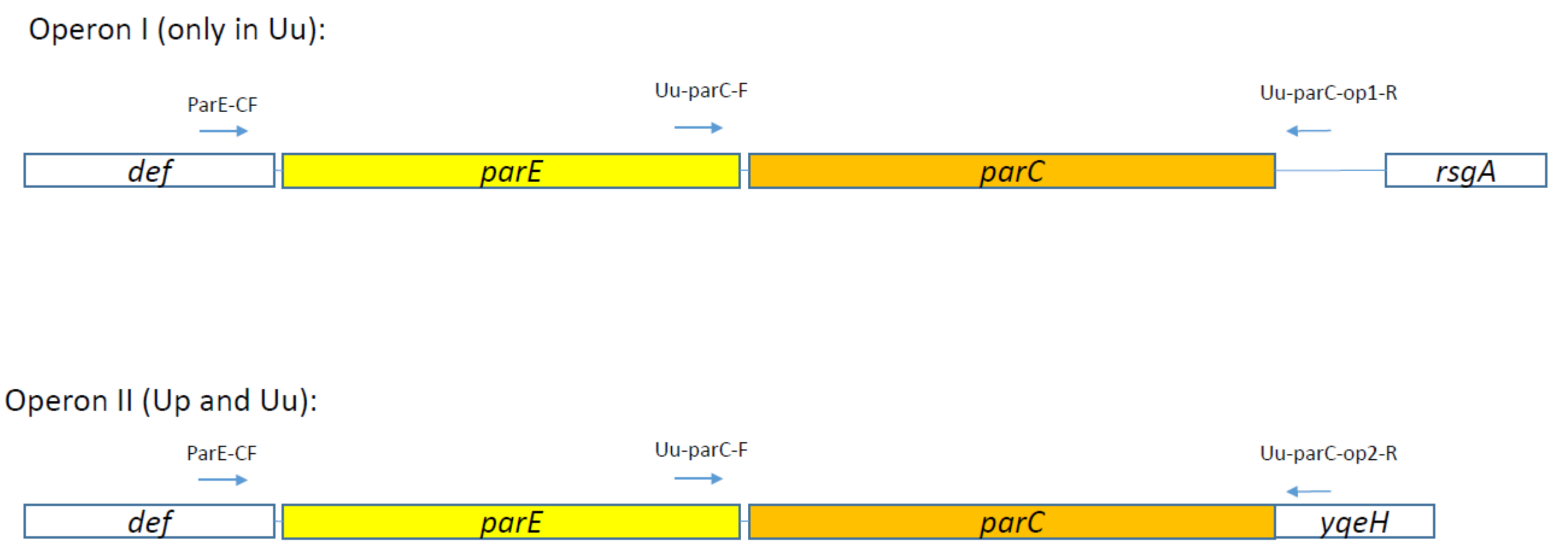

Supplementary Figure 1. *parE-parC* operons in *U. urealyticum*. The two operons were showed using ATCC strain 33699 (serovar 10, genome NC_011374) as example. The genes upstream and downstream of Operon I (561631 to 566130) and Operon II (599322 to 603814) as well as primers used to amplify the two operons were shown.

Supplementary Figure 2. MIC distributions.

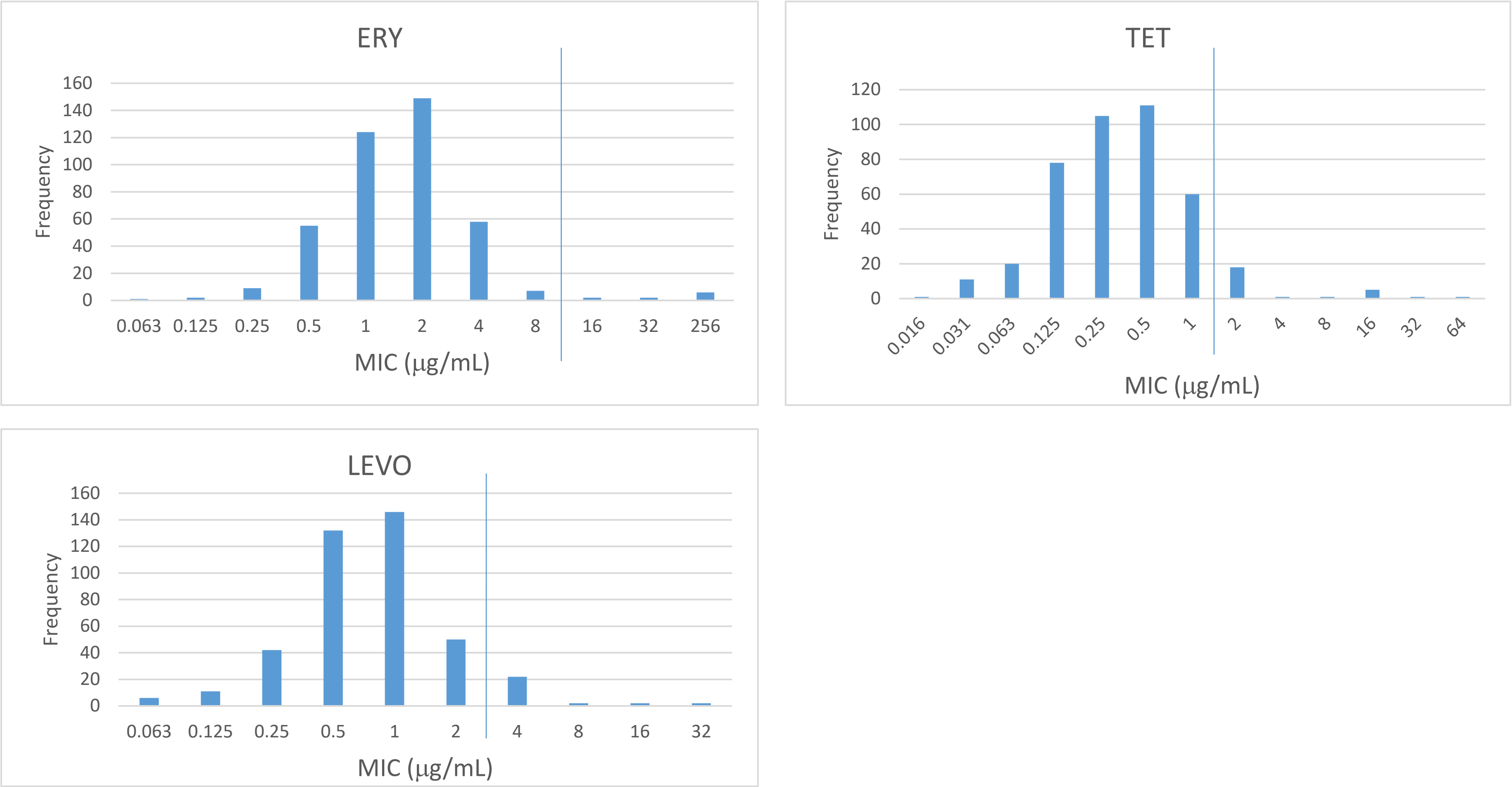

Supplementary Figure 2. MIC distributions. The distributions of MICs for erythromycin (ERY), tetracycline (TET), and levofloxacin (LEVO) were shown with the lines dividing the breakpoints for each drug.
